# Response to Antiangiogenic Therapy Is Associated with *AIMP* Protein Family Expression in Glioblastoma and Lower-Grade Gliomas

**DOI:** 10.1158/2767-9764.CRC-25-0170

**Published:** 2025-09-16

**Authors:** Humaira Noor, Yuanning Zheng, Haruka Itakura, Olivier Gevaert

**Affiliations:** 1 Stanford Center for Biomedical Informatics Research, Department of Medicine, Stanford University School of Medicine, Stanford, California.; 2 Department of Medicine, Division of Oncology, Stanford University School of Medicine, Stanford, California.; 3 Department of Biomedical Data Science, Stanford University School of Medicine, Stanford, California.

## Abstract

Glioblastoma (GBM) is a highly vascularized, heterogeneous tumor, yet antiangiogenic therapies have yielded limited survival benefits. The lack of validated predictive biomarkers for treatment response stratification remains a major challenge. Aminoacyl tRNA synthetase complex–interacting multicomplex proteins (*AIMP*) 1/2/3 have been implicated in central nervous system diseases, but their roles in gliomas remain unexplored. We investigated their association with angiogenesis and their significance as predictive biomarkers for antiangiogenic treatment response. In this multi-cohort retrospective study, we analyzed glioma samples from The Cancer Genome Atlas, Chinese Glioma Genome Atlas, REMBRANDT, Gravendeel, BELOB, and REGOMA trials, and four single-cell transcriptomic datasets. Multiomic analyses incorporated transcriptomic, epigenetic, and proteomic data. Kaplan–Meier and Cox proportional hazards models were used to assess the potential prognostic value of *AIMP*s in heterogeneous and homogeneous treatment groups. Using single-cell transcriptomics, we explored spatial and cell type–specific *AIMP2* expression in GBM. *AIMP1/2/3* expressions correlated significantly with angiogenesis across The Cancer Genome Atlas cancers. In gliomas, *AIMP*s were upregulated in tumor versus normal tissues, higher- versus lower-grade gliomas, and recurrent versus primary tumors (*P* < 0.05). Upon retrospective analysis of two clinical trials assessing different antiangiogenic drugs, we found that high-*AIMP2* subgroups had improved response to therapies in GBM [REGOMA: HR, 4.75 (1.96–11.5), *P* < 0.001; BELOB: HR, 2.3 (1.17–4.49), *P* = 0.015]. *AIMP2*-*cg04317940*methylation emerged as a clinically applicable stratification marker. Single-cell analysis revealed homogeneous *AIMP2* expression in tumor tissues, particularly in astrocyte-like cells, suggesting a mechanistic link to tumor angiogenesis. These findings provide novel insights into the role of *AIMP*s in angiogenesis, offering improved patient stratification and therapeutic outcomes in recurrent GBM.

**Significance:**

This study identifies *AIMP2* as a novel biomarker predictive of antiangiogenic treatment response in recurrent GBM. Through multiomic and single-cell analyses, *AIMP2* is shown to be upregulated in aggressive gliomas and linked to angiogenesis. Its expression and methylation status offer a clinically applicable stratification tool, enabling more personalized therapeutic approaches and improved outcomes in patients receiving antiangiogenic therapies.

## Introduction

Angiogenesis is a key pathologic hallmark of solid tumors. Among all solid tumors, glioblastomas (GBM) have the most expansive vascular systems supporting tumor growth and proliferation (1–4). A range of antiangiogenic therapies, including anti-VEGF therapies, have been largely explored in treating GBM and higher-grade astrocytoma/oligodendroglioma during the past decade. Bevacizumab, an anti-VEGF drug, was approved by FDA for the treatment of recurrent GBM in 2017 (5). Although a prolonged progression-free survival was noted, the studies failed to observe any effects on the overall survival (OS) of patients with recurrent GBM (5–8). As first-line treatment for newly diagnosed GBM, no survival benefit with the treatment of bevacizumab was observed (9, 10). Clinical trial outcomes of other antiangiogenic therapies, such as aflibercept (11), dovitinib (12), sunitinib (13), sorafenib (14), cediranib (15), imatinib (16), pazopanib (17) and marizomib (18), were not encouraging for the treatment of recurrent GBM with no observed effects on progression-free survival and/or OS. Despite the failures of antiangiogenic therapies in improving glioma survival, angiogenesis remains an important therapeutic target in this disease because of its potential to treat the highly vascularized tumor. This is substantiated by more than 40 currently on-going clinical trials of antiangiogenic drugs for GBM (clinicaltrials.gov).

It is likely that the lack of effective patient stratification and selection has contributed to the limited success of antiangiogenic therapies in GBM/other higher-grade gliomas, and identifying subgroups that may benefit from these treatments could enhance therapeutic outcomes. Currently, there are no validated biomarkers for effectively selecting patients with GBM who would benefit from antiangiogenic treatments. Thus, predictive molecular biomarkers can be crucial to patient selection, and, ultimately, success in treatment efficacy (19).

Aminoacyl tRNA synthetase (ARS) complex–interacting multi-complex proteins (*AIMP*) 1/2/3 are parts of a group of multi-functional proteins that form the ARS. The *AIMP*s are closely linked auxiliary proteins (20) playing a crucial scaffolding role in multi-tRNA synthetase complex assembly (21). The *AIMP*s have also shown to play a role in neurologic diseases and nervous system functions (22–26); however, despite their involvement in the central nervous system functions, the genomic and epigenomic roles of the *AIMP*s have not yet been explored in gliomas.

The association between *AIMP1* and angiogenesis has been established (27–29), whereas *AIMP2* may play a role in angiogenesis through modulation of the WNT/β-catenin signaling pathway (30), regulating *TRAF2 *(31) and *FUBP1* (32) that in turn affect VEGF and angiogenesis. *AIMP3* regulates p53 activation and genomic stability (33, 34) that modulate angiogenesis. Despite the relationships between *AIMP*s and angiogenesis, the genomic and epigenomic roles of *AIMP*s have not yet been studied in light of angiogenesis in cancer. Given the potential theoretical effect of *AIMP*s in glioma angiogenesis, investigating the influence of *AIMP*s in glioma angiogenesis and antiangiogenic treatment response is warranted, particularly in order to explore the potential of *AIMP*s in stratifying responders to these treatments.

In this study, we comprehensively investigated the association between *AIMP* expressions and angiogenesis gene sets across 33 cancers to uncover their link with the angiogenesis pathway. We then focused on differential multiomic expressions of *AIMP*s in glioma versus normal brain to gauge the potential impact of *AIMP* expressions in regulating antiangiogenic treatment response in glioma. We then analyze the potential prognostic roles of *AIMP*s in multi-cohort gliomas regardless of treatment received to provide rationale for retrospectively studying their potential prognostic roles in two antiangiogenic treatment-specific clinical trial cohorts. We explored *AIMP*s as predictive biomarkers for antiangiogenic treatment response in these clinical trials. To identify CpG sites that both regulate *AIMP* expression and determine treatment response, which may be used practically at the lab setting to stratify patients, we performed a comprehensive epigenetic study of *AIMP* CpGs. To provide a possible mechanistic explanation for *AIMP*s relationship with angiogenesis, we utilized four additional single-cell transcriptomic cohorts, including one spatial transcriptomic cohort, and explored *AIMP* associations with certain cell types at single-cell level. In summary, this study aims to lay a foundation for utilizing *AIMP* status as a potential biomarker for future antiangiogenic clinical trials.

## Materials and Methods

### Glioma datasets

Multiple publicly available glioma datasets were used for analysis across several sections of this study. These included multi-institutional datasets with genomic and clinical data [The Cancer Genome Atlas (TCGA), Chinese Glioma Genome Atlas (CGGA), REMBRANDT, and Gravendeel], two antiangiogenic therapy clinical trials of recurrent GBM (REGOMA and BELOB trials), one spatial transcriptomic GBM dataset (35), and three concatenated single-cell transcriptomic GBM datasets (GSE131928, GSE163108, and GSE84465). In this section, we detail the sources and data modalities for all dataset for reference.

TCGA-GBM (*n* = 152) and TCGA–low-grade glioma (LGG; *n* = 292) transcriptomic, CpG-level epigenetic and clinical data were downloaded from www.cbioportal.org. For transcriptomic data, only cases with available RNA sequencing (RNA-seq) data were selected. Although pathologic treatment response data for TCGA-GBM were not available, they were available for certain TCGA-LGG (particularly for astrocytoma subtype). Thus, bevacizumab pathologic treatment response analysis was performed on only TCGA-astrocytoma.

CGGA-GBMLGG (total *n* = 325) transcriptomic and clinical data were downloaded from www.cgga.org.cn. For transcriptomic data, only cases with available RNA-seq data were selected. Primary (*n* = 229) and recurrent gliomas (*n* = 62) were present in this dataset, unlike TCGA in which only primary tumors were predominant.

REMBRANDT-GBM (*n* = 219), REMBRANDT-LGG (*n* = 225), Gravendeel-GBM (*n* = 159), and Gravendeel-LGG (*n* = 117) transcriptomic and clinical data were downloaded from https://gliovis.bioinfo.cnio.es/. In these cohorts, transcriptomic data available were microarray. All *AIMP1/2/3* microarray data were available.

The REGOMA clinical trial (*n* = 71; refs. 36, 37) transcriptomic and clinical data were downloaded from https://www.ncbi.nlm.nih.gov/geo/query/acc.cgi?acc=GSE154041. For this dataset, the transcriptomic data available were processed with RNA-seq. The BELOB clinical trial (*n* = 112; ref. 38) transcriptomic and clinical data were downloaded from https://www.ncbi.nlm.nih.gov/geo/query/acc.cgi?acc=GSE72951. For this dataset, the transcriptomic data available were processed with microarray. Both of these clinical trials assessed antiangiogenic treatment for recurrent GBMs.

Single-cell transcriptomic cohorts analyzed included GSE131928 (24,131 cells), GSE163108 (8,252 cells), and GSE84465 (3,589 cells). Sources were https://www.ncbi.nlm.nih.gov/geo/query/acc.cgi?acc=GSE131928, https://www.ncbi.nlm.nih.gov/geo/query/acc.cgi?acc=GSE163108, and https://www.ncbi.nlm.nih.gov/geo/query/acc.cgi?acc=GSE84465, respectively.

One spatial transcriptomic cohort (*n* = 16; 69,647 spots; ref. 35) was used for spatial visualization of *AIMP* expressions (https://datadryad.org/dataset/doi:10.5061/dryad.h70rxwdmj#readme).

### Pan-cancer association between angiogenesis genes and *AIMP1/2/3*

Pan-cancer analysis to determine the association between angiogenesis pathway genes and *AIMP1/2/3* mRNA expression levels was performed on the 33 TCGA cancers. Normalized pan-cancer transcriptomic data were downloaded from the GEPIA2 platform (http://gepia2.cancer-pku.cn/). Two angiogenesis-related gene sets were used for correlation with *AIMP* expressions across TCGA cancers. First gene set was Panther pathway angiogenesis (141 genes; RRID: SCR_004869) and the gene set was downloaded from https://maayanlab.cloud/Harmonizome/gene_set/Angiogenesis/PANTHER+Pathways. The second gene set was pathway angiogenesis (76 genes; RRID: SCR_018145) and the gene set was downloaded from https://www.gsea-msigdb.org/gsea/msigdb/cards/kegg_vegf_signaling_pathway. The genes for each source have been listed in Supplementary Data File S1. For each cancer type, the *AIMP1/2/3* mRNA expression levels were correlated with the combined gene set signature mRNA expression levels. The combined gene set signature levels were generated on the GEPIA2 platform, which calculates the mean of the log_2_-transformed gene expression for each of the genes in the gene signature set for each patient. Pearson correlation coefficients were reported after adjusting for FDR with the Benjamini–Hochberg method and adjusted *P* values less than 0.05 were considered statistically significant (Supplementary Data File S2). Heatmaps were created with the Seaborn package using Python (version 3.10.9; RRID: SCR_008394).

### Multiomic differential expression of *AIMP1/2/3* in gliomas

First, transcriptomic data were analyzed to understand differential expressions of *AIMP1/2/3* in gliomas. The mRNA expression levels (RNA-seq) of *AIMP1/2/3 *in TCGA-GBM and TCGA-LGG were compared with normal brain tissue transcriptomic data. The data were sourced from the GEPIA2 platform which utilizes the UCSC Toil pipeline on TCGA and GTEx data using consistent alignment, quantification, and normalization methods. A Student *t* test comparison was performed to compare tumor versus normal groups, with *P* value < 0.05 considered statistically significant.

To compare primary versus recurrent tissues, the mRNA expression levels were analyzed in the CGGA dataset, in which sufficient primary and recurrent glioma cases were available for analysis (refer to “Glioma datasets” section). To compare expression differences between grades, the *AIMP1/2/3* mRNA expression levels in lower (WHO grade II)- and higher (WHO grade III and IV)-grade gliomas were compared in the CGGA glioma dataset. The Mann–Whitney test comparison was performed, with *P* value < 0.05 considered statistically significant.

To visualize and compare *AIMP*s at the proteomic level, we analyzed the IHC of *AIMP1/2/3* protein levels with annotated protein-level data, which were downloaded from The Human Protein Atlas (https://www.proteinatlas.org/; RRID: SCR_006710) for GBM, LGG, and normal brain tissue, and reported accordingly. The IHC slides have been presented along with annotated protein levels data for gliomas and normal brain tissue in Fig. 2B and C.

### Epigenetic regulation of *AIMP1/2/3* in gliomas

We performed a comprehensive CpG-wide analysis for *AIMP1/2/3* genes using TCGA data, in which matched transcriptomic data were available. Methylation levels (β values) for each CpG site were downloaded for *AIMP1/2/3*. CpG site–specific methylation levels of *AIMP1/2/3* were correlated with their respective gene mRNA expression levels to identify potential epigenetic regulators of gene expression in TCGA-GBM and TCGA-LGG. Pearson correlation coefficients were reported, with *P* value < 0.05 considered statistically significant. Heatmaps were created with the Seaborn package using Python (version 3.10.9; RRID: SCR_008394). Significant correlations were marked with asterisk on the heatmaps, whereas negative and positive correlations were marked with hues of blue and red, respectively.

### Prognostic effects of *AIMP1/2/3* mRNA expression and CpG-level methylation

We analyzed the potential prognostic effects of *AIMP1/2/3* mRNA expression on the OS of patients with GBM and LGG in TCGA, CGGA, REMBRANDT, and Gravendeel datasets. Survival analysis for was conducted using the Kaplan–Meier method, and the “survival” and “survminer” packages in R were utilized to assess the survival effects. Log-rank *P* value < 0.05 was considered statistically significant. We analyzed the *AIMP1/2/3* CpG-level methylation effects on the OS of TCGA-GBM and TCGA-LGG datasets using a Cox proportional hazards model. HRs with 95% confidence intervals (CI) were reported. *χ*^2^*P* values < 0.05 were considered statistically significant. Bonferroni correction was used for adjusted *P* values.

We analyzed the potential prognostic effects of specific CpG-level methylation of *AIMP1/2/3* within subgroups of bevacizumab-treated patients only in TCGA-GBM (*n* = 80) and TCGA-astroctyoma (*n* = 47) using a Cox proportional hazards model for OS and disease-free survival (DFS). Both univariate analysis and multivariate analysis adjusting for confounding factors were performed. R packages “survival” and “forestplot” were used for analysis. HRs with 95% CIs were reported. *χ*^2^*P* values < 0.05 were considered statistically significant. Bonferroni correction was used for adjusted *P* values. Only significant CpGs were visualized in forest plots. For TCGA-astroctyoma with available treatment response data (*n* = 22), we compared stable versus progressive disease using a Student *t* test (*P* value < 0.05 considered statistically significant).

### Retrospective analysis of GBM clinical trial datasets: REGOMA and BELOB

We examined the efficacies of antiangiogenic therapies in recurrent GBM clinical trials, REGOMA and BELOB. The REGOMA trial was a multicenter, open-label, randomized, controlled phase II trial (REGOMA) for investigating the effect of regorafenib in patients with recurrent GBM (*n* = 71 for whom transcriptomic data were available). The BELOB trial was a phase II, multicenter, randomized clinical trial that evaluated the efficacy of bevacizumab and lomustine (CCNU) in patients with recurrent GBM (*n* = 112 for whom transcriptomic data were available). We investigated the efficacies of these treatments in *AIMP1/2/3* high- and low-mRNA expression subgroups stratified by median expression cutoff values. In each subgroup, we performed the Kaplan–Meier method and Cox proportional hazards model analysis using IBM SPSS (RRID: SCR_002865), with *P* value < 0.05 considered significant in log-rank and *χ*^2^*P* values, respectively.

### Single-cell transcriptomic and spatial transcriptomic analysis

We previously preprocessed, normalized, and concatenated three single-cell transcriptomic cohorts: GSE131928 (*n* = 28), GSE163108 (*n* = 16), and GSE84465 (*n* = 4; ref. 39). Briefly, these datasets were merged into a unified expression matrix and underwent batch-effect normalization. We have also previously annotated the cell types in these datasets based on established marker gene expression patterns as outlined by Zheng and colleagues (39). These annotations were used for cell type–specific comparisons in this work.

The spatial transcriptomic data were processed and aligned with the corresponding single-cell data using methods outlined in Zheng and colleagues (39), in which spatial data across 23 GBM samples were batch-corrected and analyzed. Clustering of spatial transcriptomic spots and cell state assignments were carried out using consensus nonnegative matrix factorization, and spot deconvolution was performed using the reference single-cell data with Tangram (RRID: SCR_006152). Cell counts were estimated using StarDist for nuclei segmentation on matched histology images.

The Python package “scanpy” was used for spatially visualizing transformed spatial transcriptomic data for *AIMP1/2/3*. *AIMP1/2/3* levels were compared between GBM cell types [astrocyte (AC) like, oligodendrocyte progenitor cell like, mesenchymal (MES)-like 1, MES-like 2, and neural progenitor cell like] in the spatial transcriptomic cohort and the concatenated single-cell transcriptomic cohort (consisting of three datasets). Violin plots were generated using the Python “seaborn” package (version 3.10.9; RRID: SCR_008394). Kruskal–Wallis *P* value < 0.05 was considered statistically significant. Mean and median expression values were calculated for each cell type to determine cell types with highest *AIMP2* expression. Only *AIMP2 *showed significant differences across cell types in both cohorts.

### Statistical analysis

All statistical analyses were performed using R (version 4.2.2) and Python (version 3.10.9; RRID: SCR_008394). Data preprocessing and normalization were conducted as per dataset-specific protocols. The Student *t* test and Mann–Whitney tests were used for pairwise comparisons (e.g., progressive disease vs. stable disease, tumor vs. normal tissues, and primary vs. recurrent gliomas), with *P* < 0.05 considered statistically significant. Pearson correlation was used to assess associations between *AIMP1/2/3* expression and angiogenesis gene signatures, with results visualized via heatmaps using the Seaborn package.

Survival analyses were performed using the Kaplan–Meier method and Cox proportional hazards models, utilizing the “survival” and “survminer” R packages. Log-rank tests determined statistical significance (*P* < 0.05). Survival risk tables were reported in Supplementary Table S1. The median survival with 95% CIs is reported in Supplementary Table S4. Cox models were adjusted for relevant clinical covariates, reporting HRs with 95% CI. Multivariate analysis and multiple hypothesis testing were corrected using Bonferroni adjustment where applicable (Supplementary Tables S2–S4). For the REGOMA and BELOB clinical trials, treatment response was assessed using Kaplan–Meier survival analysis and Cox regression, stratified by median *AIMP* expression. IBM SPSS Statistics (version 29.0.2.0; RRID: SCR_002865) was used for these analyses, with log-rank and *χ*^2^*P* values < 0.05 deemed significant.

Single-cell and spatial transcriptomic data were analyzed using the Scanpy Python package. Kruskal–Wallis tests (*P* < 0.05) compared *AIMP *expression across GBM cell subtypes, with violin plots generated via Seaborn.

### Data availability

All data are available in the main article or supplemental files. All datasets used in this study are publicly available from previously published sources. No new datasets were generated or analyzed beyond those obtained from public repositories. Details on data sources and accession numbers are provided in the Materials and Methods section. The codes used in this article can be accessed at https://github.com/Humaira77/AIMP_Codes.

### Ethics Statement

Details of all available data are provided in the article. No institutional review board or ethics committee approval was required for this article.

## Results

### *AIMP1/2/3 *mRNA expression correlates with angiogenesis pathway across majority of TCGA cancers

**Figure 1.**
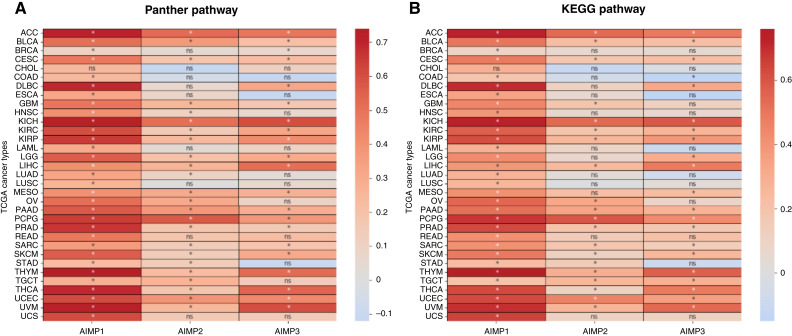
*AIMP1/2/3* correlates with angiogenesis pathway gene sets. Correlation between *AIMP1/2/3 *mRNA expression in 33 TCGA cancers and (**A**) 141 Panther pathway “angiogenesis gene set” and (**B**) 76 Kyoto Encyclopedia of Genes and Genomes (KEGG) pathway “VEGF Signaling pathway” gene set. The color map represents Pearson correlation coefficient R values. *asterisk represents statistically significant correlation (*P* < 0.05). ns represents statistically insignificant cases. ACC, Adenocortical Carcinoma; BLCA, Bladder Urothelial Carcinoma; BRCA, Breast Invasive Carcinoma; CESC, Cervical Squamous Cell Carcionoma; CHOL, Cholangiocarcinoma; COAD, Colon Adenocarcinoma; DLBC, Lymphoid Neoplasm Diffuse Large B Cell Lymphoma; ESCA, Esophageal Carcinoma; GBM, Glioblastoma Multiforme; HNSC, Head and Neck Squamous Cell Carcinoma; KICH, Kidney Chromophobe; KIRP, Kidney Renal Papillary Cell Carcinoma; LAML, Acute Myeloid Leukemia; LGG, Lower-grade Glioma; LIHC, Liver Hepatocellular Carcinoma; LUAD, Lung Adenocarcinoma; LUSC, Lung Squamous Cell Carcinoma; MESO, Mesothelioma; OV, Ovarian Serous Cystadenocarcinoma; PAAD, Pancreatic Adenocarcinoma; PCPG, Pheochromocytoma and Paraganglioma; PRAD, Prostate Adenocarcinoma; READ, Rectum Adenocarcinoma; SARC, Sarcoma; SKCM, Skin Cutaneous Melanoma; STAD, Stomach Adenocarcinoma; TGCT, Testicular Germ Cell Tumors; THCA, Thyroid Carcinoma; THYM, Thymoma; UCEC, Uterine Corpus Endometrial Carcinoma; UCS, Uterine Carcinosarcoma; UVM, Uveal Melanoma.

To establish possible associations between *AIMP*s and angiogenesis in cancer, we investigated the correlation between *AIMP1/2/3* and the angiogenesis Panther pathway and Kyoto Encyclopedia of Genes and Genomes pathway gene sets mRNA expressions across 33 TCGA cancers (Fig. 1A and B). Strikingly, *AIMP1* expression showed significant positive correlation across all cancers except CHOL. *AIMP2* and *AIMP3* expressions showed significant correlation in 24 of 33 and 21 of 33 TCGA cancers in Panther pathway analysis, respectively (Fig. 1A). In Kyoto Encyclopedia of Genes and Genomes pathway gene set analysis, *AIMP2* and *AIMP3* expressions showed significant correlation with angiogenesis gene set expressions in 20 of 33 and 21 of 33 TCGA cancers, respectively (Fig. 1B). These findings strongly suggest an association between *AIMP1/2/3* genes and angiogenesis pathways across cancers, providing rationale for investigating the role of *AIMP*s in antiangiogenesis treatment response in GBM and LGG due to *AIMP*s’ role in central nervous system functions and the increased vascularity in these tumors.

Furthermore, to demonstrate that this observed significant association is not normal tissue specific, but in fact cancer specific, we compared *AIMP* mRNA expression levels in the 33 TCGA cancers with their respective normal tissues (Supplementary Fig. S1). The *AIMP*s were significantly differentially expressed between tumor and normal tissues across various cancers (Supplementary Fig. S1). These findings suggest that *AIMP*s may be involved in common cancer pathways across multiple cancers, including angiogenesis as shown in Fig. 1, and cancer type–specific investigations of *AIMP*s are warranted.

### *AIMP* mRNA and proteins are differentially expressed in GBM and LGG tissues compared with normal brain tissue

**Figure 2.**
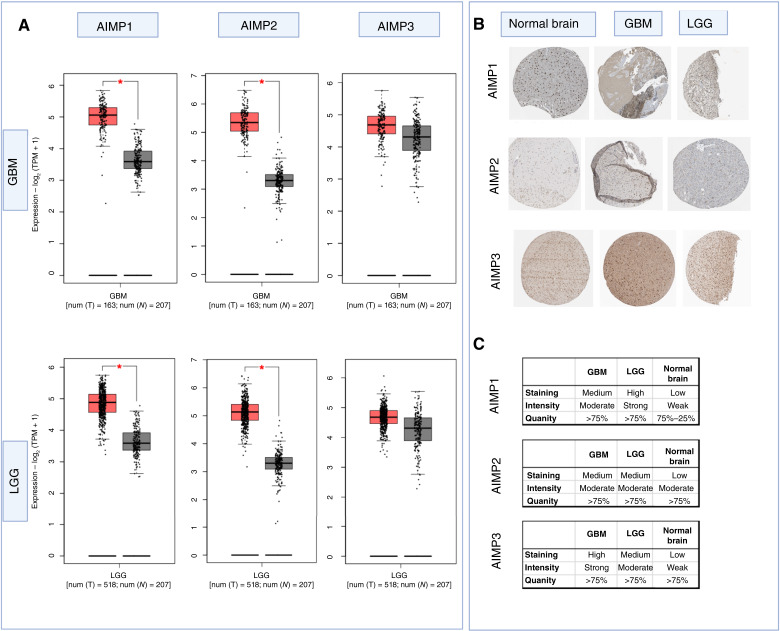
*AIMP1*, *AIMP2*, and *AIMP3* are differentially expressed in glioma tissues. *AIMP1/2/3* (**A**) mRNA (red bars: tumor samples; gray bars: normal samples) and (**B** and **C**) protein expression in TCGA-GBM, TCGA-LGG, and normal brain (GTEx) tissues. Data for (**B**) and (**C**) were collected from the Human Protein Atlas. A Student *t* test was used *, *P* < 0.05.

Furthermore, we focused on gliomas and expanded our multiomic analysis to include protein expression levels to understand the varying correlation results for gliomas in Fig. 1. First, we investigated the mRNA expression levels of *AIMP1/2/3 *in TCGA-GBM (*n* = 163), TCGA-LGG (*n* = 512) and normal brain (GTex, *n* = 207) in order to determine possible differential mRNA expression in the tumor tissue compared with normal brain tissue. We found significantly higher mRNA expression levels of *AIMP1* and *AIMP2* in both GBM and LGG compared with normal brain tissue [Fig. 2A, *P* values 0.029 (GBM-*AIMP1*), 0.015 (GBM-*AIMP2*), 0.027 (LGG-*AIMP1*), and 0.018 (LGG-*AIMP2*)]. *AIMP3* expression was comparable between the tumor tissues and normal brain tissue with no significant difference (*P* > 0.05). It should be noted that these are not paired samples from the same individuals, and although they have been pre-processed and normalized accordingly to enable comparisons, the differences in expression should be interpreted as hypothesis-generating rather than definitive. At the protein level, however, all three genes showed higher protein expression in IHC staining of GBM tissues and LGG tissues compared with normal brain tissue (Fig. 2B and C). The staining for AIMP1/2/3 in normal tissue was “low” with weak/moderate intensity, whereas the staining for GBM and LGG was consistently “high” or “medium” with strong/moderate intensity for all three proteins (Fig. 2C). This suggests that although at the mRNA level no significant difference in expression was observed for *AIMP3* while comparing tumor tissues with normal tissue, *AIMP3 *may be involved in tumorigenesis and it may be an important factor to investigate in GBM and LGG as an elevated protein expression was observed in IHC. Hence, we included *AIMP3* in the analysis of this study.

### *AIMP*s are associated with higher tumor grade and recurrence, and their effects on OS may potentially be dependent on treatment

**Figure 3.**
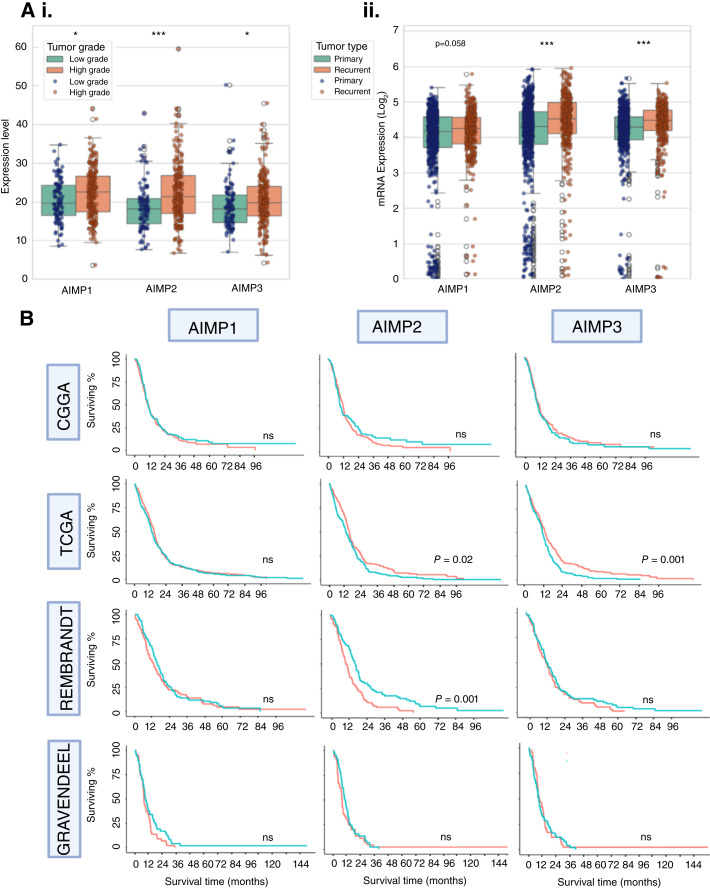
*AIMP*s are associated with tumor aggressiveness, recurrence, and prognosis. *AIMP* mRNA expression levels in (**Ai**) low-grade (II; *n* = 103) and high-grade (III and IV; *n* = 218) gliomas (*P* values 0.011, < 0.001, and 0.014 for *AIMP1*, *AIMP2*, and *AIMP3*, respectively), and (**Aii**) primary (*n* = 229) and recurrent gliomas (*n* = 62) in CGGA dataset (*P* values 0.058, *P* < 0.001, and *P* < 0.001 for *AIMP1*, *AIMP2*, and *AIMP3*, respectively). A Mann–Whitney test *P* value < 0.05 was considered statistically significant. **B,** Kaplan–Meier survival curves depicting prognostic effects of *AIMP1/2/3 *mRNA expressions in CGGA, TCGA, REMBRANDT, and Gravendeel cohorts of GBM. Red = high expression and green = low expression based on median cutoff; A log-rank *P* value < 0.05 is considered significant.

To understand the association between *AIMP*s and tumor aggressiveness, we compared *AIMP* mRNA expression levels between low-grade (grade II) and high-grade (grades II and IV) tumors (Fig. 3Ai). *AIMP1*, *AIMP2*, and *AIMP3* were significantly upregulated in high-grade tumors compared with low-grade tumors. We compared *AIMP* expression between primary and recurrent tumors to understand whether *AIMP*s are recurrence related and may be of particular importance in tumor progression (Fig. 3Aii). We found that *AIMP2* and *AIMP3* were significantly upregulated in recurrent gliomas compared with primary gliomas. There was a trend for increased *AIMP1 *expression in recurrent gliomas compared with primary gliomas (*P* = 0.058). This observation supports the association between tumor recurrence/progression and increased AIMP levels and substantiates the basis for investigating *AIMP*s particularly in recurrent GBM.

Before investigating *AIMP*s’ potential prognostic role in antiangiogenic therapy–treated patients, we analyzed their potential prognostic role in multiple glioma cohorts with heterogeneous treatment. This can potentially indicate any role *AIMP1/2/3* may play in tumor aggressiveness and progression in general, regardless of treatment. We performed survival analysis based on *AIMP1/2/3* mRNA expression levels in four GBM cohorts (TCGA *n* = 151, CGGA *n* = 220, REMBRANDT *n* = 181, and Gravendeel *n* = 155) and five LGG cohorts (TCGA-astrocytoma *n* = 193, TCGA-oligodendroglioma *n* = 99, CGGA-astrocytoma *n* = 88, REMBRANDT-oligodendroglioma *n* = 50, and REMBRANDT-astrocytoma *n* = 104). The Kaplan–Meier survival curves stratified by high- and low-AIMP1/2/3 mRNA levels are presented in Fig. 3B for GBM and Supplementary Fig. S2 for LGG.

In GBM, *AIMP1* was not found to be a significant factor affecting survival in any of the four cohorts. There were inconsistent results for *AIMP2* in TCGA and REMBRANDT cohorts, in which *AIMP2* expressions significantly affected prognosis; however, in TCGA, high expression was associated with improved survival whereas in REMBRANDT, it was associated with poorer survival (*P* < 0.05; Fig. 3B). At multivariate analysis adjusting for patient age and sex, however, *AIMP2 *was significantly associated with survival only in the TCGA cohort (Supplemetary Table S5). *AIMP3 *showed significant potential prognostic relevance in only the TCGA cohort, with high expression levels associating with improved survival in both univariate and multivariate analysis (*P* < 0.05; Fig. 3B; Supplemetary Table S5). The results for LGG were of similar patterns (Supplementary Fig. S2), with no corroborating results across cohorts.

Our analysis suggests that the roles of *AIMP* expression on survival outcomes are cohort dependent and are not consistent across multiple cohorts. A likely explanation for this is the possible heterogeneous treatment received by patients in each cohort and the possibility that *AIMP* expression potentially affects treatment response. This observation is potentially aligned with the hypothesis of our study and supports further investigations into the role of *AIMP*s in treatment response in homogeneously treated cohorts, particularly antiangiogenic therapies.

### Antiangiogenic treatment shows efficacy in highly expressed *AIMP* subgroups of GBM in homogeneously treated REGOMA and BELOB clinical trials

**Figure 4.**
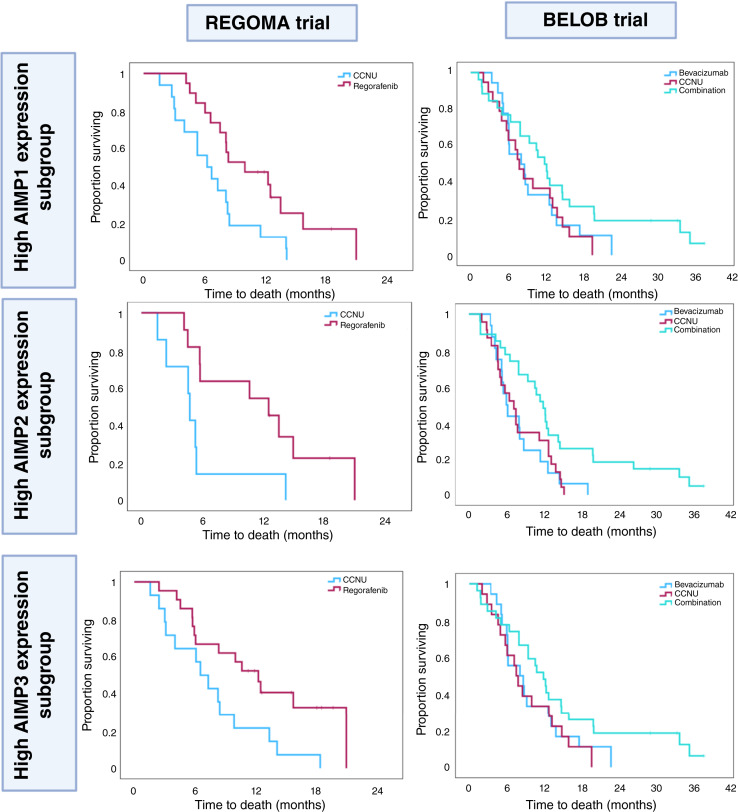
High *AIMP* mRNA expression subgroups are more responsive to antiangiogenic therapies. Kaplan–Meier survival analysis on retrospective clinical trials of recurrent GBM (REGOMA and BELOB trials). High expression sub-groups are stratified by median mRNA expressions of *AIMP1/2/3*. A log-rank *P* value < 0.05 is considered significant.

**Figure 5.**
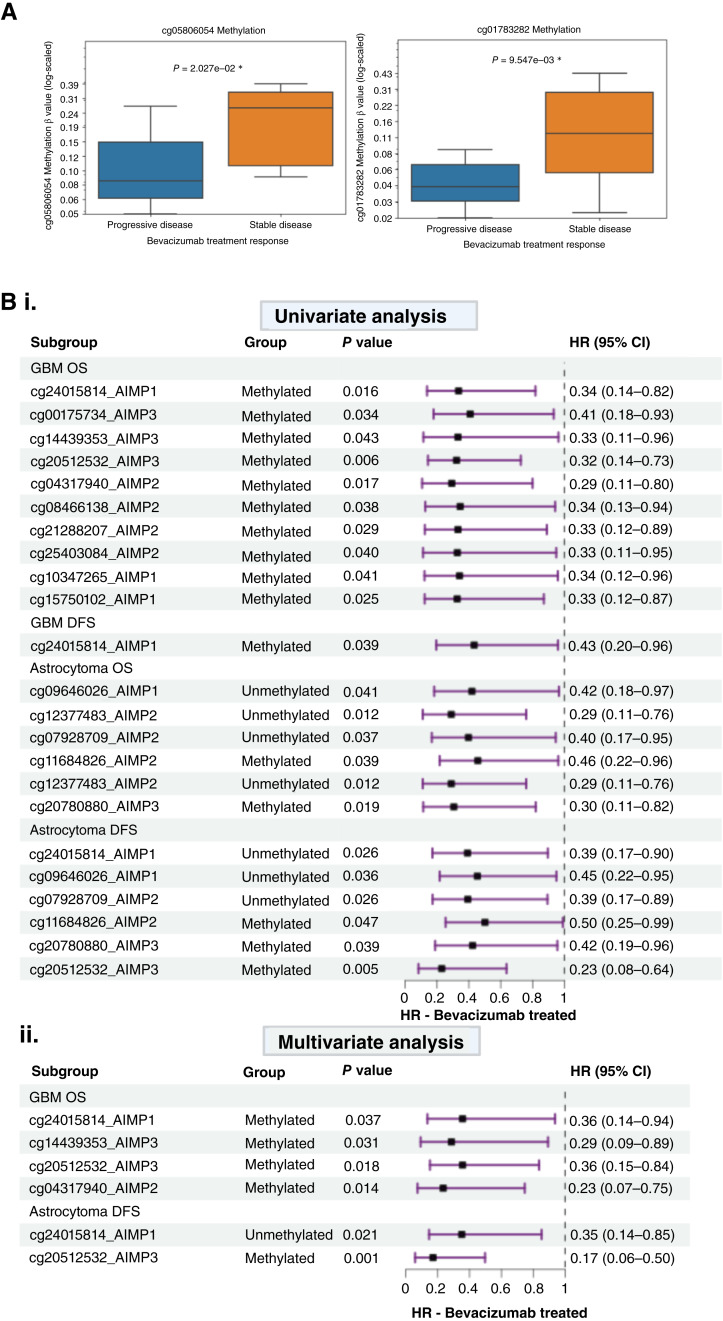
Bevacizumab treatment response is associated with specific *AIMP* CpG site methylation status in gliomas. **A,** Significant association between two CpG sites and astrocytoma (*n* = 22) pathologic response to bevacizumab treatment. A Student *t* test *P* value < 0.05 was considered significant. **B**, Forest plots depicting Cox proportional hazards model univariate (**i**) and multivariate (**ii**) analysis for the effects of *AIMP1/2/3* CpG methylation status in GBM (*n* = 27) and astrocytoma (*n* = 24).

To understand whether *AIMP*s have a role in determining antiangiogenic treatment response, we retrospectively analyzed the outcome of two GBM clinical trials with homogeneous treatment, REGOMA (36, 40) and BELOB (38), for which pretreatment transcriptomic data were publicly available. We investigated OS in patients following antiangiogenic treatments within *AIMP1/2/3*-high and -low expression sub-groups (Fig. 4) and the median survival in each group is reported for publicly available glioma datasets and the two clinical trial datasets in Supplementary Table S6. The REGOMA trial, an Italian clinical trial for patients with recurrent GBM following chemoradiotherapy, compares the efficacy of CCNU versus regorafenib (an antiangiogenic drug) monotherapy. In the original study, regorafenib was associated with significantly improved OS compared with CCNU monotherapy; however, our retrospective analysis of this cohort on the basis of *AIMP1/2/3* expression subgroups revealed that this association is only observable in *AIMP1/2/3*–high expression subgroups (*P* < 0.05; Fig. 4) and not in the low expression subgroups (*P* > 0.05; Supplementary Fig. S3). Regorafenib treatment was associated with significantly improved survival compared with CCNU treatment in high *AIMP1* [HR (95% CI), 2.41 (1.15–5.06); *P* = 0.019], high *AIMP2* [HR (95% CI), 4.75 (1.96–11.5); *P* < 0.001], and high *AIMP3* [HR (95% CI), 2.45 (1.14–5.27) *P* = 0.021] expression subgroups (Fig. 5A).

The BELOB trial was a Netherlands-based clinical trial of 148 patients with recurrent GBM following chemoradiation. The BELOB trial compares treatment with bevacizumab, CCNU, and combination therapy with the two (38). In the original BELOB study, no benefit for combination therapy or bevacizumab alone was observed; however, in our retrospective sub-group analysis, we identified significantly improved survival in patients with combination therapy compared with bevacizumab alone [HR (95% CI), 2.3 (1.17–4.49); *P* = 0.015] or CCNU alone [HR (95% CI), 1.99 (1.084–3.66); *P* = 0.026] in the *AIMP2*–high expression sub-group (Fig. 4). This was not observed in *AIMP1*– or *AIMP3*–high expression sub-groups (Fig. 4) or any of the *AIMP1/2/3*–low expression groups (Supplementary Fig. S3). This observation underscores the importance of *AIMP2* as a predictive biomarker for antiangiogenesis combination treatment response, which was not observed in the mixed group in the original study.

The results from these two retrospective analysis based on *AIMP* expression sub-groups indicate that *AIMP*s, particularly *AIMP2*, may influence response to antiangiogenic therapies such as bevacizumab and regorafenib in recurrent GBMs on which chemoradiotherapy was ineffective. The type of antiangiogenic drug was a factor in determining whether antiangiogenic monotherapy was more efficacious or there was greater benefit with CCNU combination treatment. In both cases, the denominating factor of antiangiogenic treatment addition improved patient survival in high-*AIMP2* expression subgroups of patients with recurrent GBM. These findings suggest that *AIMP* expression status may be associated with response to antiangiogenic therapy in recurrent GBM, indicating its potential as a candidate biomarker for further investigation in future clinical studies. However, it should be noted that multivariate analysis adjusting for clinical covariates such as age and sex could not be performed because of the lack of available clinical information for these two cohorts.

### Potential applicability of specific *AIMP* CpG methylation status as a predictive biomarker for antiangiogenic therapy response

As *AIMP*-high versus -low groups are relative and no threshold exists for determining potential high-expression subgroups except a relative median cutoff, we sought to identify specific *AIMP* CpGs for which methylation status may act as a practically applicable threshold for these sub-groups. This is similar to MGMT CpGs (*cg12981137*, *cg12434587*, *cg12981837*, and *cg03071809*) that determine MGMT methylation status, acting as potential predictive biomarker for chemotherapy response (41). For this, we first correlated *AIMP* mRNA expression with *AIMP* CpG methylation levels in TCGA cohorts to identify CpGs that may potentially epigenetically regulate the *AIMP*s (Supplementary Fig. S4). The significance of identifying epigenetic regulators of gene expression is well established, as evidenced by the development of numerous bioinformatics tools designed for this purpose (42–44). After identifying CpGs that significantly correlate with their respective mRNA expression levels, we performed a CpG-wide survival analysis to determine potentially prognostic CpGs regardless of treatment type (Supplementary Table S2). *AIMP2* CpG *cg11684826*, which negatively correlates with *AIMP2* expression, was a significant prognosticator for both OS and DFS in GBM. This suggests that the effects of *cg11684826* on survival may not be anti-angiogenic treatment specific, and it may not be a useful predictive biomarker although it showed significant correlation with expression. In LGG, there were no prognosticators for astrocytoma OS; however, *AIMP3-cg05806054* and *AIMP3-cg01783282* were significantly associated with astrocytoma DFS (Supplementary Table S2). These two CpG site methylation levels were also significantly lower in stable disease versus progressive disease in bevacizumab-treated astrocytoma (Fig. 5A) and may play a role in astrocytoma treatment response. However, they do not correlate with *AIMP3* mRNA expression levels in LGG, and therefore, the methylation status of these CpGs cannot be used to determine *AIMP3* expression sub-groups (Supplementary Fig. S4).

To identify antiangiogenic treatment–specific *AIMP* CpG prognosticators, we examined the OS and DFS of TCGA-GBM and TCGA-LGG for which treatment data were available for bevacizumab status. In TCGA-LGG, we only analyzed the astrocytoma subtype as there was limited bevacizumab treatment data for oligodendroglioma. We performed Cox proportional hazards model analysis at the univariate and multivariate levels (adjusting for age, sex, race, and grade) and significant results are depicted in the forest plots in Fig. 5Bi, ii. We identified four *AIMP1/2/3* CpG sites in GBM that remained significant prognosticators of OS after adjusting for age, sex, and race (*AIMP1-cg24015814*, *AIMP2-cg04317940*, *AIMP3-cg14439353*, and *AIMP3-cg20512532*). Methylation levels of these CpGs significantly correlate with their respective mRNA expression levels and may thus be useful in stratifying *AIMP*–high expression subgroups that may benefit from antiangiogenic therapies in GBM. *AIMP1-cg24015814* and *AIMP3-cg20512532* were also associated with improved DFS with bevacizumab treatment in astrocytoma at multivariate analysis (Fig. 5) and may thus be of particular importance in gliomas in general, warranting further investigations which is beyond the scope of this study. From this analysis, we report four CpG site methylations predictive of bevacizumab response that may be practically utilized at the lab setting to stratify possible responders (*AIMP1-cg24015814*, *AIMP2-cg04317940*, *AIMP3-cg14439353*, and *AIMP3-cg20512532*). Our collective results from the retrospective clinical trial analysis suggest that *AIMP2 *expression may be the strongest candidate for predicting antiangiogenesis treatment response; thus, we emphasize on the applicability of AIMP2-*cg04317940 *as a stratifying factor in bevacizumab treatment response in TCGA-GBM (*n* = 80) and TCGA-LGG (*n* = 47). Only significant results are reported (*χ*^2 ^*P* value < 0.05).

### *AIMP2* is upregulated in EGFR-related AC-like GBM cells at single-cell resolution with homogeneous spatial distribution

**Figure 6.**
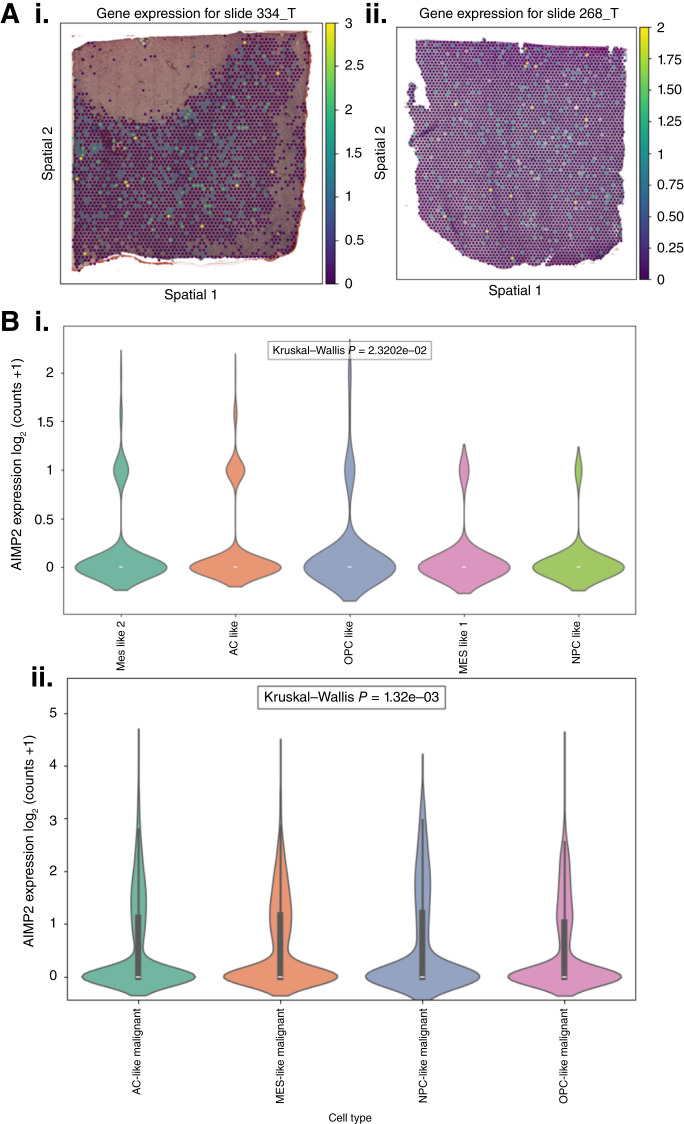
Spatial and cell type–specific expression of *AIMP2 *in GBM tumors. **A,** Spatial distribution of *AIMP2* expression across tumor tissues in two representative GBM slides (**i**: slide 334_T and **ii**: slide 268_T) analyzed using a spatial transcriptomic dataset. The heatmaps display homogeneous expression of *AIMP2* with varying intensity from low (purple) to high (yellow), indicating the absence of localized expression hotspots. **B,** Violin plots representing *AIMP2* expression across GBM cellular subtypes. **i,***AIMP2* expression in key GBM subtypes, including MES-like, AC-like, oligodendrocyte progenitor cell (OPC)–like, and neural progenitor cell (NPC)–like cells from a concatenated single-cell RNA-seq cohort (Kruskal–Wallis *P* = 2.32e−02). **ii,***AIMP2* expression across malignant GBM subtypes from a spatial transcriptomic dataset. (Kruskal–Wallis *P* = 1.32e−03).

Finally, to investigate the spatial distribution of *AIMP2* expression within GBM tumors, we analyzed a spatial transcriptomic dataset (35). This analysis revealed a homogeneous expression pattern of *AIMP2* across tumor tissues, with expression levels ranging from low or near-zero expression to high but lacking distinct regions of co-localization (Fig. 6ai, ii). The widespread expression of *AIMP2* may have significant therapeutic implications for antiangiogenic treatments as it indicates that the entire tumor—not just isolated regions—may rely on angiogenesis-driven survival mechanisms. As our results revealed that *AIMP2-*high tumors respond favorably to antiangiogenic therapy, this homogeneous expression pattern may ensure that antiangiogenic therapy may exert a more uniform therapeutic effect, potentially reducing the likelihood of therapy-resistant subpopulations emerging within the tumor. The expression patterns of *AIMP1* and *AIMP3 *were similar to those of *AIMP2 *(Supplementary Fig. S5). It may be useful to note here that with recently developed advanced computational models, *AIMP *mRNA expressions may be predicted directly from histology slides for patient stratification without the need for RNA-seq (45).

To determine the cell type specificity of *AIMP2* expression, we compared its expression levels across key GBM cellular subtypes—MES like, AC like, oligodendrocyte progenitor cell like, and neural progenitor cell like—in both the spatial transcriptomic dataset and a separate concatenated single-cell RNA-seq cohort comprising three independent GBM datasets (Fig. 6bi, ii). In both datasets, AC-like cells exhibited the highest *AIMP2* expression, followed by MES-like cells (Kruskal–Wallis *P* < 0.05). Given that AC-like cells are characterized by EGFR amplification/overexpression (46, 47), which regulates VEGF expression (48), the enrichment of *AIMP2* in this subtype may explain a possible mechanistic link between *AIMP2* expression and the tumor's angiogenic profile. This connection further supports the observed therapeutic benefit of antiangiogenic treatment in *AIMP2*-high tumors.

## Discussion

Antiangiogenesis therapies have been studied in gliomas for a long time; however, their efficacy has not been reflected through improving the OS of the patients. These therapies continue to be tested in new clinical trials to this date as a potential treatment because of the high vascularization in gliomas, particularly GBM. Currently, there is no validated predictive biomarker for stratifying the highly heterogeneous tumors for potential response to antiangiogenesis. Given the link between *AIMP1* and angiogenesis (27–29), and the potential angiogenesis regulation by *AIMP2* and *AIMP3* through modulation of the WNT/β-catenin signaling pathway (30), and p53 activation (33, 34), respectively, we explored our hypothesis of *AIMP*s playing a key role in antiangiogenesis treatment response. In this study, we show that *AIMP* expressions’ are associated with angiogenesis pathway genes. This link between *AIMP*s and angiogenesis pathway has not been previously reported; however, there have been reports of their association with immune-related pathways. Qiu and colleagues (49) reported in their pan-cancer study that higher *AIMP2* expression may be a potential biomarker for breast cancer in the context of immunotherapy response. They focused on the association between immune regulation and tumor immune microenvironment and did not delve into exploring angiogenesis pathway association. Similarly, *AIMP1* was previously reported as part of an eight-gene immune-related signature for GBM (50). As *VEGF*, a key player in angiogenesis, may modulate immune response in glioma (51), these studies support our findings of the angiogenesis pathway association with *AIMP*s. Furthermore, in a comprehensive correlation analysis between the *AIMP* genes and 5,065 different biological pathways, only two—besides the angiogenesis-related pathway—showed significant correlation with all three *AIMP* genes, underscoring their specificity to angiogenesis (Supplementary Fig. S6).

Here, we report aberrant protein expressions of *AIMP1*, *AIMP2*, and *AIMP3* in gliomas compared with normal brain and their significantly higher mRNA expression in higher-grade compared with lower-grade tumors. We also established that recurrent GBM tumors have significantly higher expression of *AIMP/2/3* compared with primary GBM tumors. These findings indicate a link among *AIMP1/2/3* and tumor aggressiveness and tumor recurrence. Thus, although the focus of this study is antiangiogenesis therapy, it should be noted that *AIMP1/2/3* inhibition may be a potential therapeutic approach that may benefit gliomas. Preclinical brain-permeable *AIMP2* inhibitors have shown some promise in Parkinson disease (52), which may be tested in glioma cell lines in future studies. Currently, there are no reported *AIMP1* and *AIMP3* inhibitors.

Through the survival analysis of four cohorts, we showed inconsistent effects of the *AIMP*s on survival in heterogeneous treatment groups, indicating that the potential prognostic impact of *AIMP1/2/3* is potentially treatment dependent, and through retrospective analysis of two recurrent GBM antiangiogenic clinical trials, we established the role of *AIMP2* as a predictive molecular biomarker for antiangiogenic treatment response. We showed that high-*AIMP2* mRNA subgroups responded better to two different antiangiogenic therapies, reflected by improved OS. Previously, in a single-institutional study, TMEM173 and FADD were reported as potential biomarkers at proteomic-level analysis (53); however, the current study is the first report of an antiangiogenic treatment biomarker at the OS level, validated by two recurrent GBM clinical trials. However, the absence of publicly available clinical data such as age and gender in these two trials limited our ability to adjust for key covariates in the survival analysis. Therefore, our findings warrant validation in well-annotated, prospective cohorts. Moreover, we acknowledge the inherent limitations and potential biases associated with retrospective analyses, including selection bias, unmeasured confounding, and variability in data quality and clinical annotations. These limitations may influence both the observed associations and their generalizability; thus, further validation in prospective cohorts is warranted.

We report the specific CpG site methylation of *AIMP2 cg04317940* as a practically applicable stratifying factor for high *AIMP* expression patient subgroups who may benefit from antiangiogenic therapy, much like specific CpG site stratifiers of *MGMT* methylation for predicting temozolomide response at the lab setting (41). However, it should be noted as a limitation that this CpG was identified based on bevacizumab treatment response in particular, and it should be validated upon availability of methylation data for other antiangiogenic treatment datasets.

Our single-cell transcriptomic analysis provides a possible explanation for the enhanced efficacy of antiangiogenic therapy observed in *AIMP2*-high tumors. *AIMP2* was homogenously distributed across GBM tumor tissues without forming localized clusters or regions of concentrated expression. This implies that the tumor may rely on angiogenesis-driven pathways for survival, potentially explaining the observed enhanced response of *AIMP2*-high tumors to antiangiogenic therapies with a more uniform therapeutic effect across the tumor. Moreover, we determined that AC-like cells, which are associated with EGFR amplification/overexpression (46, 47), display the highest *AIMP2* expression levels across cell types in two single-cell transcriptomic cohorts. It is known that EGFR regulates VEGF, which is a key player in angiogenesis (48); thus, this may be the mechanistic explanation for the role of *AIMP2* in modulating angiogenesis treatment response. However, future studies employing spatial proteomics or multiplexed immunostaining with neoangiogenic vasculature markers are warranted to more precisely characterize the spatial relationship between *AIMP* expression and angiogenic niches within GBM.

Based on our findings, we propose that utilizing *AIMP2* high expression as a predictive biomarker, stratified by methylation, for patient selection can potentially enhance therapeutic efficacy and improve outcomes in recurrent patients with GBM receiving antiangiogenic treatments. Continued validation of the biomarker in prospective studies is essential to support clinical implementation.

Future studies should aim to elucidate the mechanistic role of *AIMP2* in GBM biology, particularly in the context of angiogenesis and tumor progression. As *AIMP*s are non-enzymatic scaffold proteins within the multi-synthetase complex (among other within the larger multi-subunit ARS complex), the biological impact of *AIMP* expression may depend not only on their absolute abundance but also on their interactions with other synthetase components. It remains to be determined whether changes in *AIMP2* expression actively contribute to disease phenotypes or are secondary to broader dysregulation in tumor biology. Investigating how relative abundance of *AIMP2* affects the structural integrity or signaling outputs of the ARS complex may offer insights into its functional relevance. Further functional studies are needed to delineate the regulatory or structural roles of* AIMP2* in glioma pathophysiology and to determine whether it has actionable potential as a therapeutic target.

## Supplementary Material

Supplementary Table S1 Risk-tables for BELOB and REGOMA trials, and CGGA, TCGA, REMBRANDT and GRAVENDEEL survival analysis

Supplementary Table S4 Results of Cox Proportional Hazards multivariate analysis of AIMP CpG methylation with adjusted p-values

Supplementary Table S3 Results of Cox Proportional Hazards univariate analysis of AIMP CpG methylation with adjusted p-values

Supplementary Table S2 Prognostic AIMP1/2/3 CpG-sites in TCGA GBM and TCGA Astrocytoma

Supplementary Table S5 Cox proportional hazards model results at multivariate analysis (TCGA and REMBRANDT)

Supplementary Table S6 Median survival with 95% confidence interval for AIMP groups vs GBM datasets

Supplementary Figure S1 Pan-cancer comparison of AIMP1/2/3 mRNA expression levels between tumor (TCGA) versus normal tissue (GTEx).

Supplementary Figure S2 Kaplan-Meier Survival Curves depicting prognostic effects of AIMP1/2/3 mRNA expressions in CGGA, TCGA, and REMBRANDT cohorts of gliomas

Supplementary Figure S3 Kaplan-Meier survival analysis on retrospective clinical trials of recurrent GBM (REGOMA and BELOB trials).

Supplementary Figure S4 Correlation between specific AIMP1/2/3 CpG site methylation levels and their respective mRNA expression levels

Supplementary Figure S5 Spatial distribution of AIMP1 and AIMP3 expression across tumor tissues in two representative GBM slides

Supplementary Figure S6 AIMP1/2/3 significantly correlates with only two pathways other than angiogenesis in a comprehensive analysis of 5065 biological pathways.

Supplementary data file 1 list of panther pathway and kegg pathway genes.

Supplementary data file 2 Pan-Cancer correlation between AIMP1/2/3 and KEGG/Panther pathway genes
