# Supplementary material for: Response to Antiangiogenic Therapy Is Associated with AIMP Protein Family Expression in Glioblastoma and Lower-Grade Gliomas
Source: Cancer Res Commun. 2025 Sep 16;5(9):1651–63. doi: 10.1158/2767-9764.CRC-25-0170 (PMC12438089; doi:10.1158/2767-9764.CRC-25-0170)
Supplement: Supplementary Table S1 — Risk-tables for BELOB and REGOMA trials, and CGGA, TCGA, REMBRANDT and GRAVENDEEL survival analysis [file crc-25-0170_supplementary_table_s1_suppst1.docx]

**Supplementary Table S1:** Risk-tables for BELOB and REGOMA trials, and CGGA, TCGA, REMBRANDT and GRAVENDEEL survival analysis

| **Belob AIMP1 high** |  |  |  |  |  |
| --- | --- | --- | --- | --- | --- |
| **Time (months)** | **0** | **12** | **24** | **36** | **48** |
| Bevacizumab | 18 | 5 | 0 | 0 | 0 |
| CCNU | 19 | 6 | 0 | 0 | 0 |
| Combination | 26 | 12 | 4 | 0 | 0 |
| **Belob AIMP2 high** |  |  |  |  |  |
| Bevacizumab | 16 | 3 | 0 | 0 | 0 |
| CCNU | 23 | 7 | 0 | 0 | 0 |
| Combination | 27 | 13 | 4 | 0 | 0 |
| **Belob AIMP3 high** |  |  |  |  |  |
| Bevacizumab | 18 | 5 | 0 | 0 | 0 |
| CCNU | 18 | 5 | 0 | 0 | 0 |
| Combination | 27 | 12 | 4 | 0 | 0 |
| **Regoma AIMP1 high** |  |  |  |  |  |
| Lomustine | 16 | 1 | 0 | 0 | 0 |
| Regorafenib | 19 | 6 | 0 | 0 | 0 |
| **Regoma AIMP2 high** |  |  |  |  |  |
| Lomustine | 14 | 0 | 0 | 0 | 0 |
| Regorafenib | 21 | 10 | 0 | 0 | 0 |
| **Regoma AIMP3 high** |  |  |  |  |  |
| Lomustine | 14 | 2 | 0 | 0 | 0 |
| Regorafenib | 21 | 8 | 0 | 0 | 0 |
| **CGGA AIMP1** |  |  |  |  |  |
| **Time (months)** | 0 | 50 | 100 |  |  |
| High | 140 | 5 | 1 |  |  |
| Low | 138 | 9 | 3 |  |  |
| **CGGA AIMP2** |  |  |  |  |  |
| High | 140 | 7 | 1 |  |  |
| Low | 138 | 7 | 3 |  |  |
| **CGGA AIMP3** |  |  |  |  |  |
| High | 143 | 8 | 1 |  |  |
| Low | 135 | 6 | 3 |  |  |
| **TCGA AIMP1** |  |  |  |  |  |
| High | 185 | 8 | 1 |  |  |
| Low | 187 | 7 | 1 |  |  |
| **TCGA AIMP2** |  |  |  |  |  |
| High | 183 | 9 | 1 |  |  |
| Low | 189 | 6 | 1 |  |  |
| **TCGA AIMP3** |  |  |  |  |  |
| High | 186 | 12 | 2 |  |  |
| Low | 186 | 3 | 0 |  |  |
| **REMBRANDT AIMP1** |  |  |  |  |  |
| High | 88 | 5 | 1 |  |  |
| Low | 93 | 9 | 0 |  |  |
| **REMBRANDT AIMP2** |  |  |  |  |  |
| High | 91 | 3 | 1 |  |  |
| Low | 90 | 11 | 0 |  |  |
| **REMBRANDT AIMP3** |  |  |  |  |  |
| High | 91 | 5 | 0 |  |  |
| Low | 90 | 9 | 1 |  |  |
| **GRAVENDEEL AIMP1** |  |  |  |  |  |
| High | 44 | 0 | 0 |  |  |
| Low | 47 | 1 | 1 |  |  |
| **GRAVENDEEL AIMP2** |  |  |  |  |  |
| High | 45 | 1 | 1 |  |  |
| Low | 46 | 0 | 0 |  |  |
| **GRAVENDEEL AIMP3** |  |  |  |  |  |
| High | 42 | 1 | 1 |  |  |
| Low | 49 | 0 | 0 |  |  |
