# Supplementary material for: Response to Antiangiogenic Therapy Is Associated with AIMP Protein Family Expression in Glioblastoma and Lower-Grade Gliomas
Source: Cancer Res Commun. 2025 Sep 16;5(9):1651–63. doi: 10.1158/2767-9764.CRC-25-0170 (PMC12438089; doi:10.1158/2767-9764.CRC-25-0170)
Supplement: Supplementary Table S4 — Results of Cox Proportional Hazards multivariate analysis of AIMP CpG methylation with adjusted p-values [file crc-25-0170_supplementary_table_s4_suppst4.docx]

**Supplementary Table S4:** Results of Cox Proportional Hazards multivariate analysis of AIMP CpG methylation with adjusted p-values

| **Subgroup** | **Group** | **est** | **low** | **hi** | **se** | **p-value** | **adjusted p-value** |
| --- | --- | --- | --- | --- | --- | --- | --- |
| **GBM Overall Survival** |  |  |  |  |  |  |  |
| cg24015814_AIMP1 | Methylated | 0.356 | 0.135 | 0.937 | 0.494 | 0.037 | 0.037 |
| cg14439353_AIMP3 | Methylated | 0.286 | 0.092 | 0.893 | 0.581 | 0.031 | 0.037 |
| cg20512532_AIMP3 | Methylated | 0.356 | 0.151 | 0.837 | 0.436 | 0.018 | 0.032 |
| cg04317940_AIMP2 | Methylated | 0.234 | 0.073 | 0.747 | 0.593 | 0.014 | 0.032 |
| **Astrocytoma Disease-Free Survival** | |  |  |  |  |  |  |
| cg24015814 | Unmethylated | 0.351 | 0.145 | 0.853 | 0.453 | 0.021 | 0.032 |
| cg20512532 | Methylated | 0.17 | 0.058 | 0.497 | 0.548 | 0.001 | 0.006 |
