# Supplementary material for: Response to Antiangiogenic Therapy Is Associated with AIMP Protein Family Expression in Glioblastoma and Lower-Grade Gliomas
Source: Cancer Res Commun. 2025 Sep 16;5(9):1651–63. doi: 10.1158/2767-9764.CRC-25-0170 (PMC12438089; doi:10.1158/2767-9764.CRC-25-0170)
Supplement: Supplementary Table S3 — Results of Cox Proportional Hazards univariate analysis of AIMP CpG methylation with adjusted p-values [file crc-25-0170_supplementary_table_s3_suppst3.docx]

**Supplementary Table S3:** Results of Cox Proportional Hazards univariate analysis of AIMP CpG methylation with adjusted p-values

| **Subgroup** | **Group** | **est** | **low** | **hi** | **se** | **p-value** | **adjusted p-value** |
| --- | --- | --- | --- | --- | --- | --- | --- |
| **GBM Overall Survival** |  |  |  |  |  |  |  |
| cg24015814_AIMP1 | Methylated | 0.335 | 0.137 | 0.818 | 0.456 | 0.016 | 0.043 |
| cg00175734_AIMP3 | Methylated | 0.408 | 0.178 | 0.933 | 0.422 | 0.034 | 0.043 |
| cg14439353_AIMP3 | Methylated | 0.331 | 0.114 | 0.963 | 0.545 | 0.043 | 0.043 |
| cg20512532_AIMP3 | Methylated | 0.322 | 0.143 | 0.726 | 0.415 | 0.006 | 0.043 |
| cg04317940_AIMP2 | Methylated | 0.292 | 0.106 | 0.8 | 0.515 | 0.017 | 0.043 |
| cg08466138_AIMP2 | Methylated | 0.345 | 0.126 | 0.942 | 0.513 | 0.038 | 0.043 |
| cg21288207_AIMP2 | Methylated | 0.331 | 0.123 | 0.89 | 0.505 | 0.029 | 0.043 |
| cg25403084_AIMP2 | Methylated | 0.327 | 0.112 | 0.949 | 0.544 | 0.04 | 0.043 |
| cg10347265_AIMP1 | Methylated | 0.341 | 0.121 | 0.959 | 0.528 | 0.041 | 0.043 |
| cg15750102_AIMP1 | Methylated | 0.326 | 0.122 | 0.871 | 0.501 | 0.025 | 0.043 |
| **GBM Disease-Free Survival** |  |  |  |  |  |  |  |
| cg24015814_AIMP1 | Methylated | 0.433 | 0.195 | 0.96 | 0.406 | 0.039 | 0.044 |
| **Astrocytoma Overall Survival** |  |  |  |  |  |  |  |
| cg09646026_AIMP1 | Unmethylated | 0.419 | 0.182 | 0.966 | 0.425 | 0.041 | 0.041 |
| cg12377483_AIMP2 | Unmethylated | 0.289 | 0.11 | 0.76 | 0.492 | 0.012 | 0.036 |
| cg07928709_AIMP2 | Unmethylated | 0.397 | 0.167 | 0.946 | 0.443 | 0.037 | 0.041 |
| cg11684826_AIMP2 | Methylated | 0.456 | 0.216 | 0.962 | 0.381 | 0.039 | 0.041 |
| cg12377483_AIMP2 | Unmethylated | 0.289 | 0.11 | 0.76 | 0.492 | 0.012 | 0.036 |
| cg20780880_AIMP3 | Methylated | 0.305 | 0.113 | 0.82 | 0.505 | 0.019 | 0.038 |
| **Astrocytoma Disease-Free Survival** | |  |  |  |  |  |  |
| cg24015814_AIMP1 | Unmethylated | 0.391 | 0.171 | 0.896 | 0.423 | 0.026 | 0.0467 |
| cg09646026_AIMP1 | Unmethylated | 0.453 | 0.216 | 0.95 | 0.378 | 0.036 | 0.046 |
| cg07928709_AIMP2 | Unmethylated | 0.393 | 0.172 | 0.894 | 0.42 | 0.026 | 0.046 |
| cg11684826_AIMP2 | Methylated | 0.501 | 0.253 | 0.991 | 0.348 | 0.047 | 0.047 |
| cg20780880_AIMP3 | Methylated | 0.423 | 0.188 | 0.956 | 0.416 | 0.039 | 0.046 |
| cg20512532_AIMP3 | Methylated | 0.229 | 0.083 | 0.636 | 0.52 | 0.005 | 0.039 |
