# Supplementary material for: Response to Antiangiogenic Therapy Is Associated with AIMP Protein Family Expression in Glioblastoma and Lower-Grade Gliomas
Source: Cancer Res Commun. 2025 Sep 16;5(9):1651–63. doi: 10.1158/2767-9764.CRC-25-0170 (PMC12438089; doi:10.1158/2767-9764.CRC-25-0170)
Supplement: Supplementary Table S2 — Prognostic AIMP1/2/3 CpG-sites in TCGA GBM and TCGA Astrocytoma [file crc-25-0170_supplementary_table_s2_suppst2.docx]

**Supplementary Table S2 : Prognostic AIMP1/2/3 CpG-sites in TCGA GBM and TCGA Astrocytoma**

|  |  | **GBM OS** |  |  |  |  |
| --- | --- | --- | --- | --- | --- | --- |
|  | coef | Hazard Ratio | se(coef) | z | p-value | Adjusted p-value |
| **AIMP2** |  |  |  |  |  |  |
| cg11684826 | 2.33 | 10.35 | 1.137 | 2.055 | 0.0398 | 0.0398 |
|  |  | **GBM DFS** |  |  |  |  |
| **AIMP2** |  |  |  |  |  |  |
| cg11684826 | 3.36 | 29.00 | 1.35 | 2.49 | 0.0127 | 0.0381 |
|  |  | **Astrocytoma DFS** |  |  |  |  |
| **AIMP3** |  |  |  |  |  |  |
| cg05806054 | 3.15 | 23.54 | 1.17 | 2.69 | 0.0069 | 0.0381 |
| cg01783282 | 2.50 | 12.18 | 1.21 | 2.06 | 0.0393 | 0.0398 |
