# Supplementary material for: Response to Antiangiogenic Therapy Is Associated with AIMP Protein Family Expression in Glioblastoma and Lower-Grade Gliomas
Source: Cancer Res Commun. 2025 Sep 16;5(9):1651–63. doi: 10.1158/2767-9764.CRC-25-0170 (PMC12438089; doi:10.1158/2767-9764.CRC-25-0170)
Supplement: Supplementary Table S5 — Cox proportional hazards model results at multivariate analysis (TCGA and REMBRANDT) [file crc-25-0170_supplementary_table_s5_suppst5.docx]

**Supplementary Table S5:** Cox proportional hazards model results at multivariate analysis

| **TCGA multivariate analysis adjusting for age and sex covariates** | | | | | |
| --- | --- | --- | --- | --- | --- |
|  | **Hazard Ratio** | | **95% confidence intervals** | | **p-value** |
| **AIMP2 group** | 0.645 | | 0.464-0.896 | | 0.009 |
| **AIMP3 group** | 0.671 | | 0.478-0.943 | | 0.021 |
|  | |  | |  |  |
| **REMBRANDT multivariate analysis adjusting for age and sex covariates** | | | | | |
|  | Hazard Ratio | | **95% confidence intervals** | | **p-value** |
| **AIMP2 group** | 1.18 | | 0.813-1.712 | | 0.384 |
