# Supplementary material for: Response to Antiangiogenic Therapy Is Associated with AIMP Protein Family Expression in Glioblastoma and Lower-Grade Gliomas
Source: Cancer Res Commun. 2025 Sep 16;5(9):1651–63. doi: 10.1158/2767-9764.CRC-25-0170 (PMC12438089; doi:10.1158/2767-9764.CRC-25-0170)
Supplement: Supplementary Table S6 — Median survival with 95% confidence interval for AIMP groups vs GBM datasets [file crc-25-0170_supplementary_table_s6_suppst6.docx]

**Supplementary Table S6:** Median survival with 95% confidence interval for AIMP groups vs GBM datasets

|  | **Median survival - 95% confidence interval (months)** | | | | | |
| --- | --- | --- | --- | --- | --- | --- |
|  | **AIMP1 low** | **AIMP1 high** | **AIMP2 low** | **AIMP2 high** | **AIMP3 low** | **AIMP3 high** |
| **CGGA** | 11.3 (9.5-12.1) | 11.8 (9.5-12.1) | 10 (7.8-12.25) | 12.5 (8.75-12.8) | 10.9 (7.7-11.2) | 12.4 (7.9-13.1) |
| **TCGA** | 12.6 (10.5 -14) | 14.7 (14.1-15.7) | 12.3 (9.6-14) | 14.7 (12.2-15.7) | 12.9 (10.5-14) | 15 (12.3-16.6) |
| **REMBRANDT** | 15.5 (13.1-16.9) | 12.75 (8.3-14.2) | 17.2 (15.3-19.6) | 11.8 (9.6-15.3) | 14.45 (10.9-16.3) | 14 (9.4-16.2) |
| **GRAVENDEEL** | 9.1 (6-13.4) | 7.8 (5.8-9.6) | 10.3 (6.1-11.5) | 7.7 (4.6-8.4) | 8.5 (5-9.6) | 9.1 (7.3-11.5) |
|  | **Median survival - 95% confidence interval (months)** | | | | | |
|  | **BELOB** | | | **REGOMA** | | |
|  | **Bevacizumab** | **CCNU** | **Combination** | **Lomustine** | **Regorafenib** | |
| **AIMP1 High** | 8.181 (3.1-13.2) | 7.885 (6.1-9.7) | 11.926 (9.7-14.1) | 6.23 (3.5-8.9) | 9.93 (4.6-15.2) | |
| **AIMP2 High** | 6.078 (4.7-7.4) | 7.261 (5.2-9.3) | 11.926 (9.5-14.3) | 4.73 (4.3-5.1) | 12.43 (4.7-20.1) | |
| **AIMP3 High** | 8.181 (3.1-13.2) | 7.589 (6.2-8.9) | 11.926 (9.1-14.7) | 6.5 (4.3-8.7) | 12.2 (8.9-15.5) | |
