# Supplementary material for: Response to Antiangiogenic Therapy Is Associated with AIMP Protein Family Expression in Glioblastoma and Lower-Grade Gliomas
Source: Cancer Res Commun. 2025 Sep 16;5(9):1651–63. doi: 10.1158/2767-9764.CRC-25-0170 (PMC12438089; doi:10.1158/2767-9764.CRC-25-0170)
Supplement: Supplementary Figure S1 — Pan-cancer comparison of AIMP1/2/3 mRNA expression levels between tumor (TCGA) versus normal tissue (GTEx). [file crc-25-0170_supplementary_figure_s1_suppsf1.docx]

**Supplementary Figure 1**


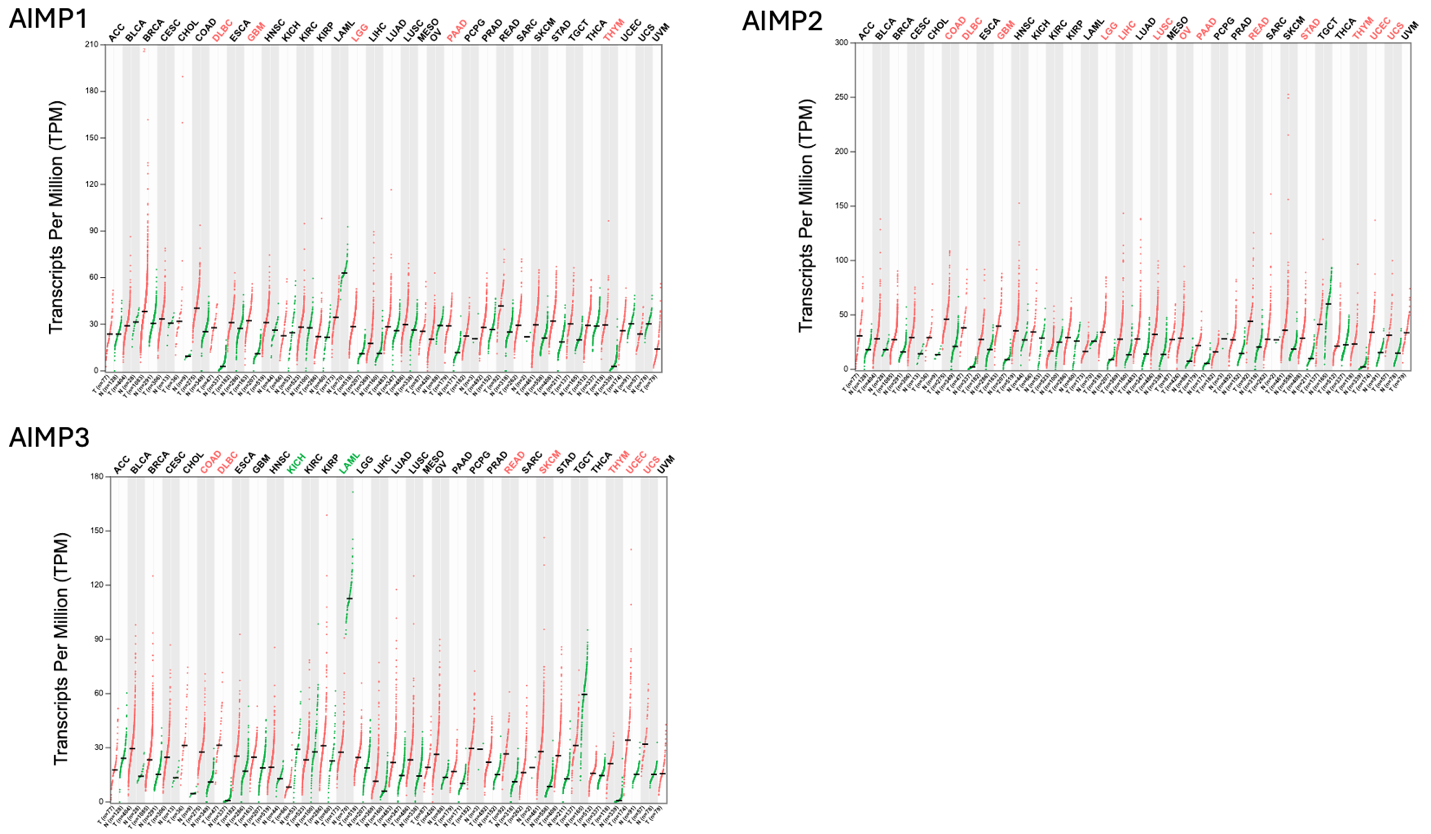


**Supplementary Figure S1.** Pan-cancer comparison of AIMP1/2/3 mRNA expression levels between tumor (TCGA) versus normal tissue (GTEx). Red label indicates significant association with higher expression in the tumor; Green label indicates significant association with higher expression in the normal tissue (p<0.05).
