# Supplementary material for: Response to Antiangiogenic Therapy Is Associated with AIMP Protein Family Expression in Glioblastoma and Lower-Grade Gliomas
Source: Cancer Res Commun. 2025 Sep 16;5(9):1651–63. doi: 10.1158/2767-9764.CRC-25-0170 (PMC12438089; doi:10.1158/2767-9764.CRC-25-0170)
Supplement: Supplementary Figure S2 — Kaplan-Meier Survival Curves depicting prognostic effects of AIMP1/2/3 mRNA expressions in CGGA, TCGA, and REMBRANDT cohorts of gliomas [file crc-25-0170_supplementary_figure_s2_suppsf2.docx]

**Supplementary Figure S2**


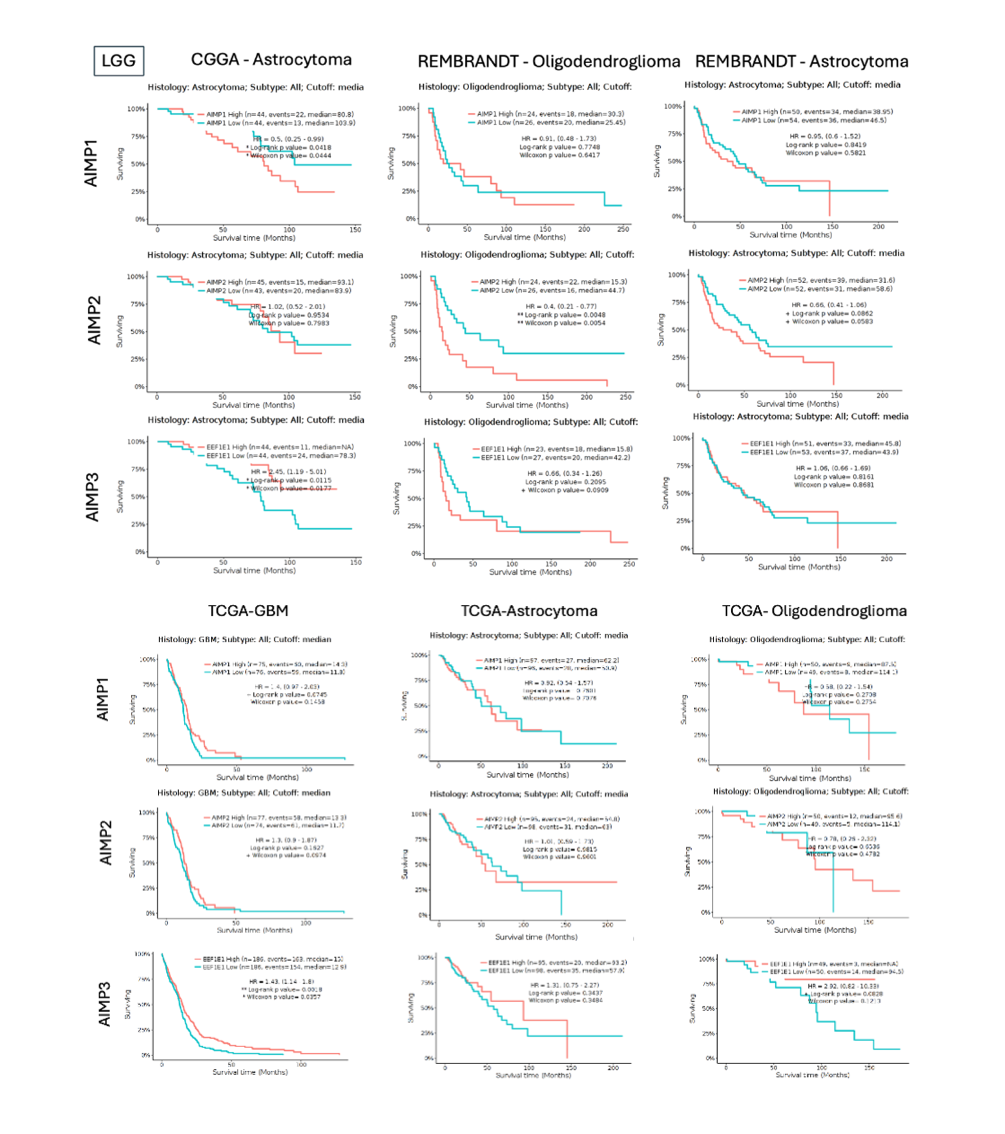


**Supplementary Figure S2.** Kaplan-Meier Survival Curves depicting prognostic effects of AIMP1/2/3 mRNA expressions in CGGA, TCGA, and REMBRANDT cohorts of gliomas. Red=high-expression green=low-expression based on median-cutoff; log-rank p-value<0.05 is considered significant.
