# Supplementary material for: Response to Antiangiogenic Therapy Is Associated with AIMP Protein Family Expression in Glioblastoma and Lower-Grade Gliomas
Source: Cancer Res Commun. 2025 Sep 16;5(9):1651–63. doi: 10.1158/2767-9764.CRC-25-0170 (PMC12438089; doi:10.1158/2767-9764.CRC-25-0170)
Supplement: Supplementary Figure S3 — Kaplan-Meier survival analysis on retrospective clinical trials of recurrent GBM (REGOMA and BELOB trials). [file crc-25-0170_supplementary_figure_s3_suppf3.docx]

**Supplementary Figure S3**

**
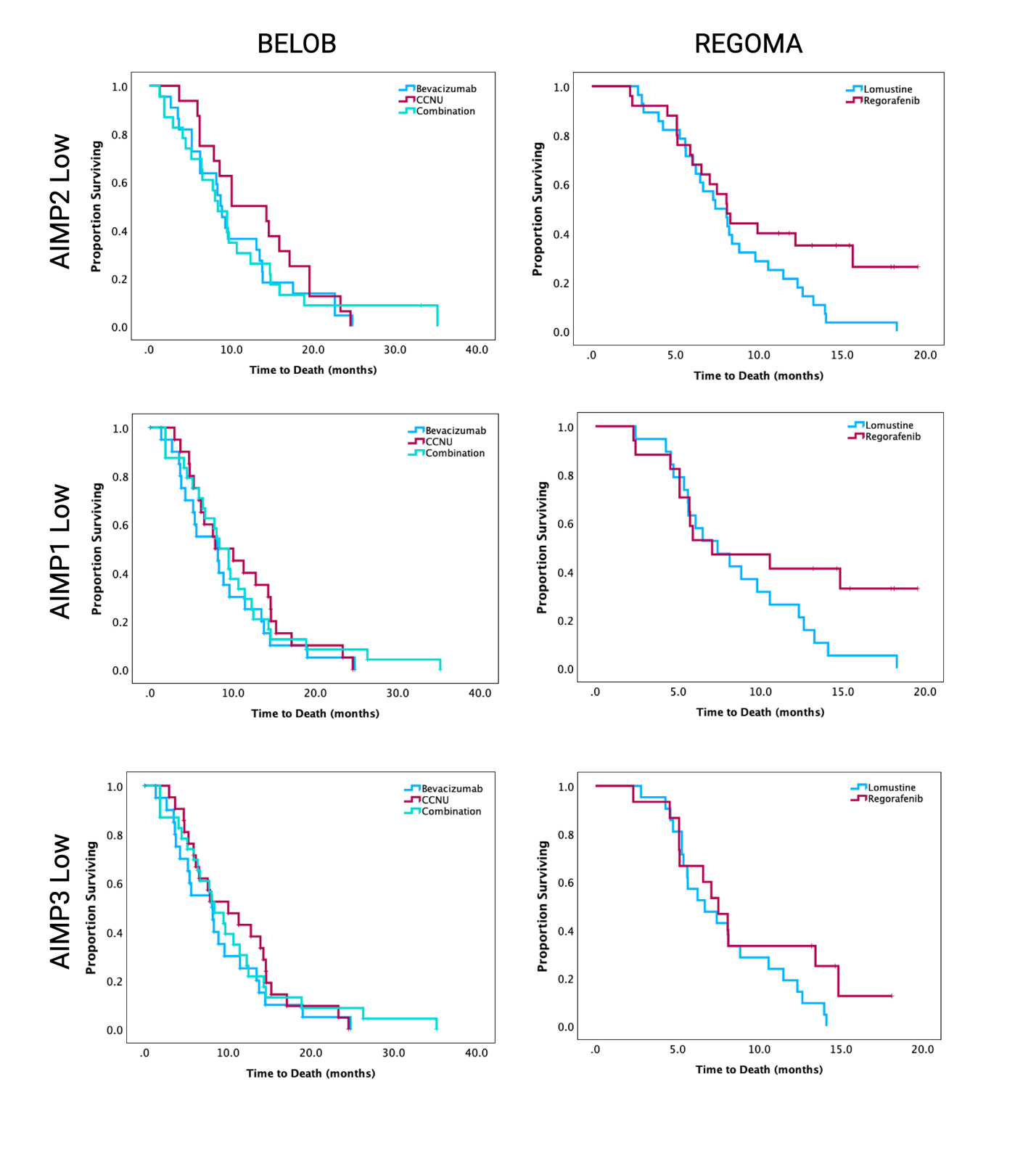
**

**Supplementary Figure S3. Low AIMP mRNA expression subgroups are not responsive to anti-angiogenic therapies.** Kaplan-Meier survival analysis on retrospective clinical trials of recurrent GBM (REGOMA and BELOB trials). Low expression sub-groups are stratified by median mRNA expressions of AIMP1/2/3. Log-rank p-value<0.05 is considered significant.
