# Supplementary material for: Response to Antiangiogenic Therapy Is Associated with AIMP Protein Family Expression in Glioblastoma and Lower-Grade Gliomas
Source: Cancer Res Commun. 2025 Sep 16;5(9):1651–63. doi: 10.1158/2767-9764.CRC-25-0170 (PMC12438089; doi:10.1158/2767-9764.CRC-25-0170)
Supplement: Supplementary Figure S4 — Correlation between specific AIMP1/2/3 CpG site methylation levels and their respective mRNA expression levels [file crc-25-0170_supplementary_figure_s4_suppsf4.docx]

**Supplementary Figure S4**


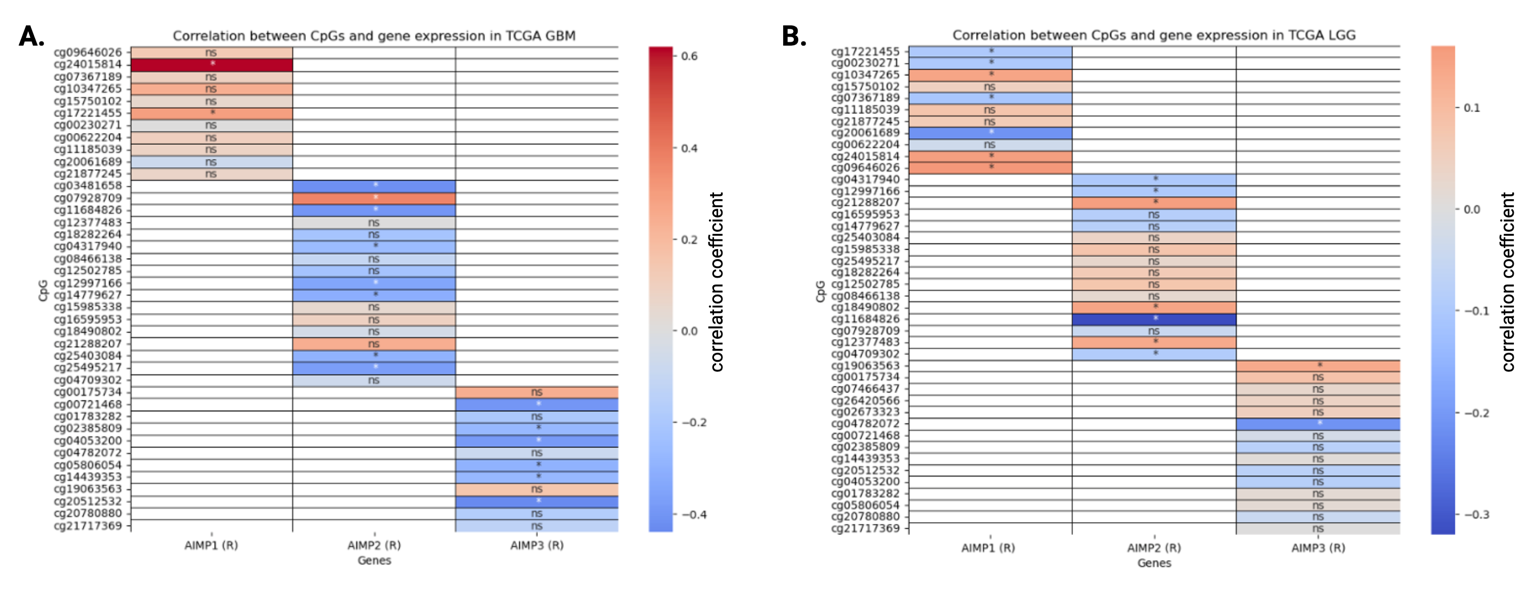


**Supplementary Figure S4.** Correlation between specific AIMP1/2/3 CpG site methylation levels and their respective mRNA expression levels in **(A)** TCGA-GBM and **(B)** TCGA-LGG. *asterisk represents significant correlation (p-value<0.05). The colormap indicates blue hues=negative correlation and red hues=positive correlation.
