# Supplementary material for: Response to Antiangiogenic Therapy Is Associated with AIMP Protein Family Expression in Glioblastoma and Lower-Grade Gliomas
Source: Cancer Res Commun. 2025 Sep 16;5(9):1651–63. doi: 10.1158/2767-9764.CRC-25-0170 (PMC12438089; doi:10.1158/2767-9764.CRC-25-0170)
Supplement: Supplementary Figure S5 — Spatial distribution of AIMP1 and AIMP3 expression across tumor tissues in two representative GBM slides [file crc-25-0170_supplementary_figure_s5_suppsf5.docx]

**Supplementary Figure S5**

**
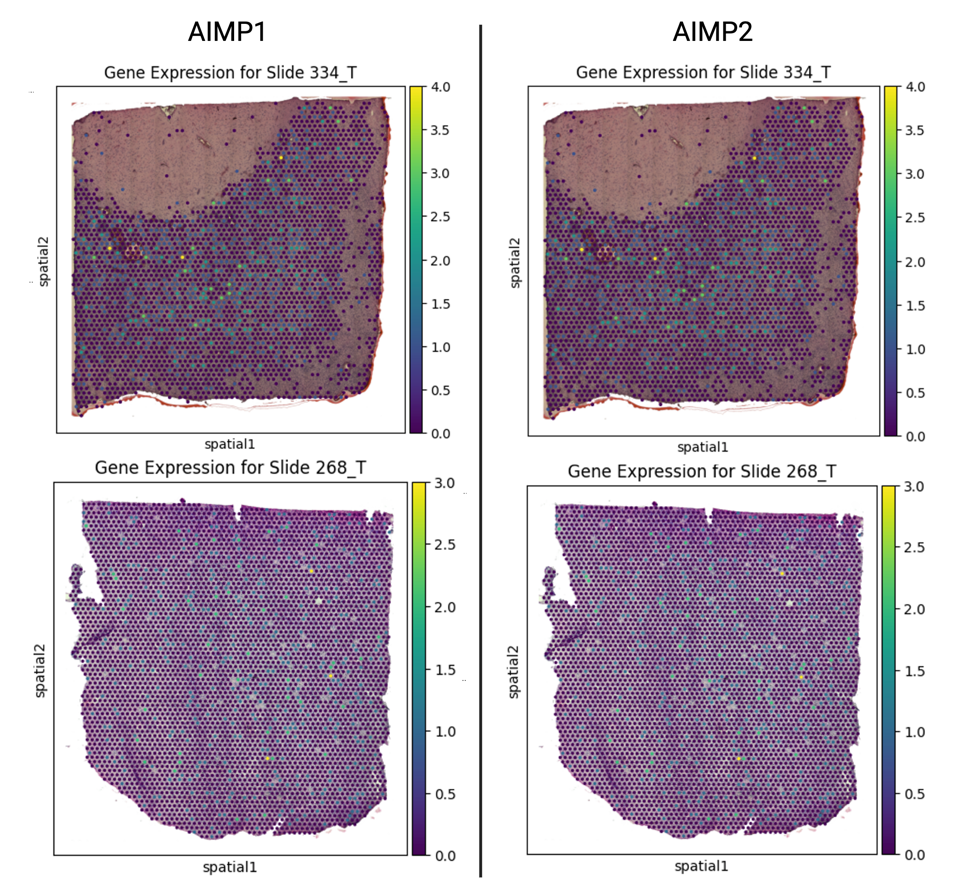
**

**Supplementary Figure S5. Spatial expression of AIMP1 and AIMP3 in glioblastoma (GBM) tumors.** Spatial distribution of AIMP1 and AIMP3 expression across tumor tissues in two representative GBM slides (i: Slide 334_T, ii: Slide 268_T) analyzed using a spatial transcriptomic dataset. The heatmaps display homogeneous expressions of AIMP1 and AIMP3 with varying intensity from low (purple) to high (yellow), indicating the absence of localized expression hotspots.
