# Supplementary material for: Response to Antiangiogenic Therapy Is Associated with AIMP Protein Family Expression in Glioblastoma and Lower-Grade Gliomas
Source: Cancer Res Commun. 2025 Sep 16;5(9):1651–63. doi: 10.1158/2767-9764.CRC-25-0170 (PMC12438089; doi:10.1158/2767-9764.CRC-25-0170)
Supplement: Supplementary Figure S6 — AIMP1/2/3 significantly correlates with only two pathways other than angiogenesis in a comprehensive analysis of 5065 biological pathways. [file crc-25-0170_supplementary_figure_s6_suppsf6.docx]

**Supplementary Figure S6**

**
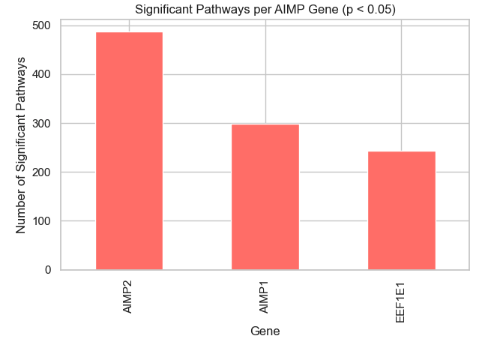

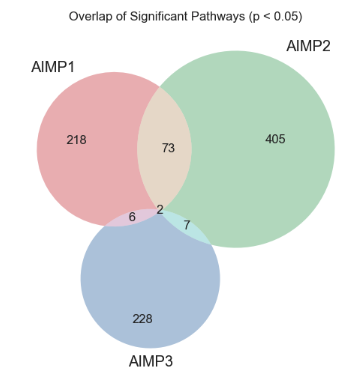
**

**Supplementary Figure S6. AIMP1/2/3 significantly correlates with only two pathways other than angiogenesis in a comprehensive analysis of 5065 biological pathways.** Number of PARADIGM pathways significantly associated with AIMP1, AIMP2 and AIMP3/EEF1E1 out of a total of 5065 biological pathways analyzed (left). Venn diagram depicting shared significantly correlated PARADIGM pathways between AIMP1/2/3 (right).
